# Pharmacists’ perceptions and attitudes toward drug importation into the State of Florida

**DOI:** 10.1186/s40545-021-00381-0

**Published:** 2021-12-02

**Authors:** John B. Hertig, Jade M. Jochem, Allissa M. Long

**Affiliations:** 1 Butler University College of Pharmacy & Health Sciences, 4600 Sunset Ave., Indianapolis, IN 46208 USA; 2 Massachusetts College of Pharmacy and Health Sciences, Boston, MA USA

**Keywords:** Drug importation, Substandard, Falsified, Drug pricing, Drug adulteration, Pharmaceutical

## Abstract

**Background:**

The Department of Health and Human Services and the Food and Drug Administration released the Safe Importation Action Plan in July 2020 detailing methods to import medicines from Canada to combat increasing drug costs. In November 2020, Florida became the first state in the United States to create and propose an importation plan from Canada. This study examines the proposal submitted by Florida, Florida pharmacists’ perceptions of the program on patient safety, and Florida pharmacists’ thoughts on the pharmacy operational impact.

**Methods:**

This was a cross-sectional study utilizing an electronic questionnaire sent to pharmacist members of the Florida Pharmacy Association. The survey incorporated closed-ended and open-ended questions. The results from the study were reported and analyzed through descriptive statistics, qualitative and quantitative data.

**Results:**

Two-hundred and forty-four pharmacists responded to the survey. Of those respondents, 25% stated they had no knowledge about Florida’s drug importation plan. Less than 12% of respondents stated they would trust the safety and quality of imported medicines. Seventy percent of pharmacists expressed concerns regarding the changes required in pharmacy operations to increase medicine safety. About half of the respondents questioned whether this plan would promote cost-savings as intended.

**Conclusion:**

Florida pharmacists believe the drug importation plan does not address all aspects of patient and medicine safety and expressed concerns regarding logistical operations of a pharmacy. This article highlights those concerns and acts as a summons to action.

**Supplementary Information:**

The online version contains supplementary material available at 10.1186/s40545-021-00381-0.

## Background

Prescription medicine costs continue to rise in the United States (US) and present a significant barrier for patients to receive care. Medicine prices in the US are 2.56 times higher than other countries [1]. This number is influenced by the cost of brand-name medicines; few, if any, price restrictions; and payer involvement (pharmacy benefit managers, insurance companies, government, etc.) in the US [1, 2]. Notably, the cost of generic medicines were 0.84 times lower in the US compared to other countries [1]. Despite generic medicines accounting for 84% of the prescription drug volume, generic medicine use represents only 12% of prescription drug spending in the US [1]. Experts contend the cost of prescription medicines is not projected to lower in the next few years, especially since drug spending in the US has increased by 76% between 2000 to 2017 [3].

To combat the raising drug costs in the US, the concept of “drug importation” or re-importation has become increasingly popular with federal and state policy makers. Drug importation is the sanctioned practice of obtaining prescription medicines from outside the US and importing them back to consumers. The concept of drug importation is not new, however, as it has historically occurred in states along the US–Canadian border, where individuals travel into Canada, visit a licensed physician, and obtain a prescription that will be filled from a legitimate Canadian pharmacy. This commonplace practice has been used to take advantage of lower drug prices in Canada [4]. Based on a RAND study, Canada’s drug prices were 46% of the US in 2018. However, generic medicines were more expensive in Canada than the US [1]. In November 2020, a final rule to implement a provision of the Federal, Food, Drug, and Cosmetic Act (section 804(b) through (h) of the FD&C Act) went into effect to allow importation of certain prescription medicines from Canada. This permits states, Indian tribes, pharmacists, and wholesalers to submit importation program proposals to the Food and Drug Administration (FDA) [5].

Several states, most notably Florida, Colorado, and Vermont, are at different stages in the implementation of importation of medicines from Canada. Of those states, Florida has completely developed and submitted “Florida’s Canadian Prescription Drug Importation Program” to the Department of Health and Human Services (HHS) [6]. This plan will permit importation of medicines for HIV/AIDS, asthma, chronic obstructive pulmonary disease, and diabetes (among others) to highlight disease states that have the highest associated prescription drug costs [7].

The Florida’s Canadian Prescription Drug Importation Program includes relabeling and repackaging the product with the ultimate destination of the pharmacy [7]. Because of this, pharmacists play a significant role in patient safety in the context of this drug importation plan. Responsibilities of pharmacists include determining how wholesalers protect their drug supply, limiting or eliminating purchases from secondary wholesalers, and not purchasing of high-risk medicines among wholesalers [8]. Incorporating these checks and balances into the already demanding workflow of a pharmacy environment has the potential to disrupt operations and increase the risk of medicine errors [8].

Given Florida’s drug importation plan does not adequately address the possibility of increased errors as part of a comprehensive patient safety plan, and pharmacists play an extremely important role in patient safety, this study is designed to investigate the perception of Florida pharmacists regarding the state’s importation plan [9]. The primary objective of this study was to determine how pharmacists in Florida perceive the drug importation plan and what apprehensions they may have about the state’s plan.

## Methods

To achieve the primary research objective, a 27-question online survey tool was created using Qualtrics^XM^ (Provo, UT). The survey was designed to collect information regarding pharmacist participants’ demographics, baseline knowledge of drug importation, and potential concerns with dispensing imported medicines from their pharmacy (Additional file 1). Given the limited pharmacist-specific research on drug importation and the novel study design, there were no standardized and validated tools or surveys to adapt at the time of this study. Therefore, this survey was created by one author, sent to experts to pilot, and feedback from these experts was used to refine the tool. This survey incorporated multiple-choice and free response options to allow participants freedom to express their opinions.

The study was voluntary, with invitations to participate distributed to pharmacist members of the Florida Pharmacy Association in November 2020 with a completion date required in December 2020 to give participants 1 month to complete the survey. One reminder e-mail was distributed during the data collection phase. All participants that responded to the survey, even if they did not fully complete the survey, were included in the analysis. No participants were excluded for any reason, unless they did not consent to the survey. To ensure adequate number of responses, a required sample size of at least 195 participants was calculated using a 95% confidence level to represent a pharmacist population of 20,200 in Florida for each question (error ± 7%) [10]. Results from the survey were analyzed and reported using descriptive statistics.

This research was determined to be exempt from full Institutional Review Board (IRB) review and was approved through the Butler University IRB.

## Results

### Participants

Of the pharmacist members e-mailed the survey tool, 1326 were delivered. Out of the 1326 e-mails that were delivered, 605 pharmacists opened the survey tool (open rate of 45.6%). In total, 244 pharmacists agreed to participate (response rate of 40.3%). Of those that agreed, 218 continued to answer the first question. The majority of the respondents identified as male (64.3%). Most respondents had at least a bachelor’s degree (62.3%) and then a Doctor of Pharmacy (PharmD) degree (28.4%). Participants primarily worked in a community pharmacy environment (35.4%) or hospital pharmacy (10.9%), though there was representation in almost all fields of pharmacy. A majority of the pharmacists graduated from pharmacy programs during or prior to the year 1987 (62.2%).

### Knowledge of drug importation

**Fig. 1:**
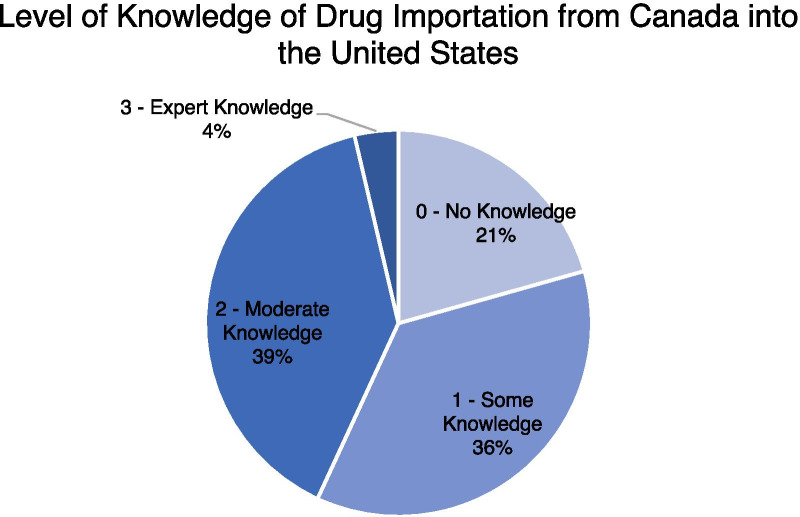
Baseline knowledge of drug importation

Participants noted that they had limited baseline importation of drug importation in general. Over one fifth (20.6%) of respondents stated that they had no knowledge of drug importation from Canada into the US. Over 35% stated that they only had some knowledge of drug importation. This totals less than half of the respondents stating that they felt they had moderate or expert knowledge in the topic (Fig. 1). Respondents stated that their knowledge of drug importation ranged from personal research, pharmacy organizations, mentors, schooling, and through media outlets.

### Concerns regarding dispensing of imported medicines

**Fig. 2:**
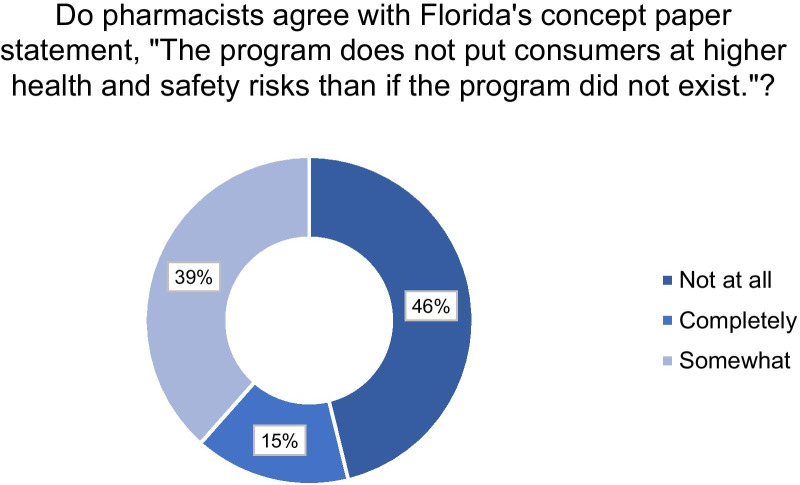
Pharmacists thoughts on overall safety of Florida’s drug importation plan

One-third of respondents would consider participating in an importation plan within their pharmacy. Less than 8% of participants would definitely fill a prescription from Canada over a US generic medicine. Furthermore, less than 20% of participants stated they would feel very confident in the safety of imported medicines and 72% of participants stated they have concerns with importing medicines. Specifically, 58% of respondents believed that there would not be adequate monitoring and safety of medicines imported from Canada. Over 46% of participants did not agree with the statement from Florida’s proposal that “the program does not put consumers at higher health and safety risks than if the program did not exist.” For more detailed information, refer to Fig. 2.

**Fig. 3:**
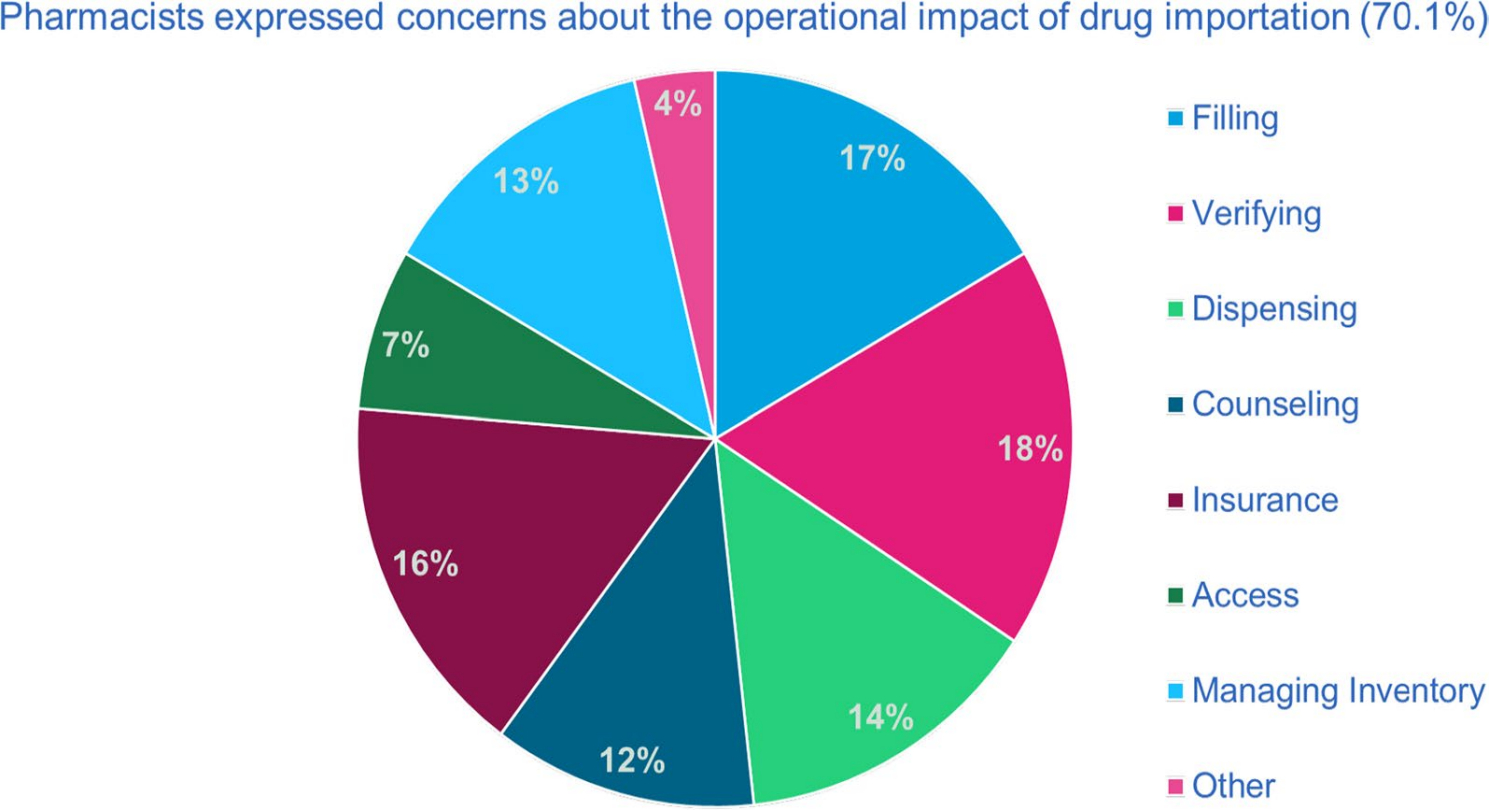
Pharmacy operational conflicts

Pharmacists also expressed concerns regarding the operational impact of drug importation (70.1%). Participants identified that all operational responsibilities of a pharmacy would be interrupted, with verifying, filling prescriptions, and processing through insurances being the most challenging (Fig. 3). Patient access to medicines was the operation that was the least identified to be impacted by drug importation (7%). Participants were concerned about the product visibly changing and the confusion that would cause patients (79.5%).

In addition to questioning the quality and safety of the medicines imported from Canada, participants doubted that the drug importation would cause medicines competition with the US market (45.7%) and that importing medicines would not lead to lowering the cost of medicines in the US (57.6%).

### Open-response sections

The survey was designed to allow respondents to include additional information with certain questions to adequately capture pharmacists’ apprehensions with the drug importation plan. One such question (Additional file 1, question 8) prompted respondents to include any concerns they had with Florida’s drug importation plan. The most common responses included patient safety; safety and quality of medicines; whether the plan would truly lead to cost savings and how insurance companies would be incorporated; and true availability of medicines from Canada. Other notable responses encompassed questionable efficacy, purity, and bioequivalence of the medicines from Canada. Pharmacists also were concerned about whether they would be liable for the medicines if there were safety issues.

Respondents indicated additional operational conflicts they foresaw with the implementation of the drug importation plan. Participants questioned how providers would be educated on the drug importation plan and how much additional time would need to be spent notifying them of the switch to a Canadian medicine. Respondents also expressed concerns about dispensing a medicine with a label that could include a different identifying features and language.

Participants were encouraged to include any additional thoughts they had on the drug importation plan at the end of the survey (Additional file 1, question 27). In addition to the concerns already expressed in other questions, some respondents identified past examples of counterfeit drugs in the US and believed that importing medicines from Canada would be no different than current processes. Others expressed optimism that importing medicines from Canada would yield improved quality controls, promote patient safety, and increase cost savings.

## Discussion

The results of this survey are one of the first data sets available after the submission of Florida’s Canadian Prescription Drug Importation Program. Assessing pharmacists’ perceptions and apprehensions is necessary to develop policies and protocols that address patient and medicine safety. Understanding operational barriers pharmacists may anticipate with drug importation will help direct pharmacy leaders to make changes as necessary.

Use of an online survey tool was beneficial to obtain pharmacists’ perceptions in a timely and efficient manner. Both the open rate and response rate for the survey were close to or above average compared to other surveys [11]. Participants that responded represented a wide range of pharmacists with varying baseline knowledge of drug importation. Respondents were engaged and answered the open-response prompts throughout the survey.

Finding reliable, consistent information regarding drug importation and Florida’s Canadian Prescription Drug Importation Program was difficult. With the submission of this proposal, access to this information should be made more readily available whether through pharmacy organizations, continuing education programs, or information from state legislators or the Board of Pharmacy directly to licensed pharmacists. If health care professionals are not properly educated on the program and have difficulty accessing resources to educate themselves, how can they guarantee that the patients will have all the information they need to make informed decisions about the medicines they are taking?

Pharmacists acknowledged that they had concerns for patient and medicines safety. Most notable were the concerns regarding overall quality, safety, and efficacy from the medicines that would be imported from Canada. With increased availability of medicine from Canada, there is the concern that patients may look elsewhere to import medicines as well. Organizations and other healthcare stakeholders have expressed concerns regarding use of the internet to purchase prescription medicines from illegal online pharmacies [12, 13]. In addition, one respondent discussed that the drug importation proposal states testing of medicines will be done by a qualified laboratory but there is no strict definition and it is unclear whether testing will be provided by the US or Canada. Florida’s proposal states that medicines manufactured by the same companies for the US and Canada will not undergo any laboratory testing [7]. Many participants had apprehensions regarding whether the medicines received from Canada would be adulterated or counterfeit—and rightly so. The repackaging and relabeling plan described in the drug importation plan includes the creation of a new label combining the information included on the Canadian and US drug label. With repackaging and relabeling, there is an increased risk of medicine errors occurring. Furthermore, the drug characteristics from medicines imported from Canada will not be the same as the drug identifiers in the US [7].

True cost-savings of the drug importation program were also discussed. Respondents expressed doubt in the plan to truly impact the cost of medicines available in the US. In the plan, all cost-savings are centered on decreased prices of medicines. However, the plan does not discuss the cost of partnerships required to implement the plan, the cost of routine laboratory testing, and the cost of repackaging and relabeling prescription drugs [7, 14]. For a more accurate cost-savings estimate, these expenses need to be considered for a net cost-savings. Multiple participants in the survey questioned how to handle insurance claims and this was not addressed in Florida’s proposal. This will need to be acknowledged prior to implementing the drug importation plan.

Accessibility of medicines was identified by respondents as a benefit of the drug importation plan. However, a Canadian Minister of Health has expressed concerns that drug exportation will create drug shortages for the citizens of Canada. Because of this, an order was passed preventing the dispensing of a medicine to a citizen outside of Canada unless it will not cause or exacerbation a drug shortage and evidence behind why it would not cause a shortage [15]. With the addition of this provision in Canada, this brings into question how much access US citizens will have to medicines at a lower cost.

As with any survey-based research study, there are limitations to consider. Pharmacists that were not members of the Florida Pharmacy Association were unable to participate in the survey. That said, the research did enroll a sufficient sample of participants. Utilizing an online survey instrument has the benefits of being easy to administer and allows increased access to respondents, but there is the consideration that this survey was not developed from previously validated tools due to the novelty of the information being investigated. This survey was also limited in scope to Florida, which may limit extrapolation across the US, but this approach did allow the investigators to focus the instrument to Florida’s importation plan.

## Conclusion

Providing cost-effective access to medicine is a national imperative in the US. Medicine costs continue to rise, which decreases patient access to quality care. It is the responsibility of both legislators and health care professionals to develop unique policy solutions to decrease the burden of health care costs safely. Drug importation has been proposed as an answer; however, it must be implemented with caution. As currently proposed, state plans including the Florida plan, do not adequately ensure medicine and patient safety at each step in the process. The subsequent operational impacts may also jeopardize any cost-savings projected. Incorporating pharmacists, along with the health care community and other stakeholders, into the decision-making process would help ensure patient safety is not unintentionally sacrificed as a result of policy.

## Supplementary Information

**Additional file 1.** Survey tool questions.

## Data Availability

The data sets used and/or analyzed during the current study are available from the corresponding author on reasonable request. The survey tool is available as supplementary material.
