# Supplementary material for: Pharmacists’ perceptions and attitudes toward drug importation into the State of Florida
Source: J Pharm Policy Pract. 2021 Dec 2;14:101. doi: 10.1186/s40545-021-00381-0 (PMC8638520; doi:10.1186/s40545-021-00381-0)
Supplement: Supplementary file 1 — Additional file 1. Survey tool questions. [file 40545_2021_381_MOESM1_ESM.docx]

**ADDITIONAL INFORMATION**

Additional 1: Survey Tool Questions

1. What is your level of knowledge of drug importation from Canada to the United States?
   1. No knowledge
   2. Some knowledge
   3. Moderate knowledge
   4. Expert knowledge
2. If you selected some, moderate or expert knowledge of drug importation, how did you learn about drug importation? (Select all the apply)
   1. Personal research
   2. Online database or organization
   3. Mentor
   4. Throughout your academic course work
   5. Other – please explain
3. Is your pharmacy currently participating in drug importation?
   1. Yes
   2. No
4. If no, would you consider participating in an importation plan in your pharmacy?
   1. Yes
   2. No
5. How likely would you fill a prescription with a drug from Canada over a generic drug, knowing that there is not much difference in price between the two?
   1. Extremely likely
   2. Somewhat likely
   3. Neither likely nor unlikely
6. Florida is implementing training requirements to prove electronic documentation is accurately being complied with. In your opinion, how helpful will this be?
   1. Not helpful at all
   2. Somewhat helpful
   3. Very helpful
7. How confident are you or would you be when dispensing exported medications that they are safe?
   1. Not confident at all
   2. Somewhat confident
   3. Very confident
8. Do you have any concerns?
   1. Yes
   2. No
9. If yes, what concerns do you have? [open-ended]
10. Do you trust the safety and quality of these foreign medications that you have been receiving and accepting into your pharmacy?
    1. Not at all
    2. Somewhat
    3. Very
11. Do you believe exporting drugs from Canada will cause competition between the US drug market and cause them to lower their drug prices?
    1. Yes
    2. No
12. Will importation have the intended impact of lowering US drug costs?
    1. Yes
    2. No
13. It is known that there is a concern for importation of counterfeit medications into the US, do you believe we can adequately monitor and ensure the safety of the medications we are importing from Canada?
    1. Yes
    2. No
14. Do you believe quarterly evaluations are enough to ensure the safety and evaluate the medications on the drug importation plan?
    1. Yes
    2. No
15. Florida’s drug importation concept paper states, “…the packaging may be different (e.g., blister packs instead of plastic bottles) and the labeling will be different in may respects (e.g., foreign language, warnings, indications for use, etc.).” Based on this information do you believe this would interfere with typical pharmacy operations?
    1. Yes
    2. No
16. If yes, which operations do you see conflicts with, select all that apply:
    1. Filling
    2. Verifying
    3. Dispensing
    4. Counseling
    5. Insurances
    6. Access
    7. Managing inventory
    8. Other – please explain
17. Patients often find comfort in being able to recognize their medication by the indicated characteristic, such as color of the medication, or markings, ect. Knowing that Canadian medications look different, do you have or would you have any reservations about confusion with patients?
    1. Yes
    2. No
18. Florida’s current drug importation concept paper states “The Program does not put consumers at higher health and safety risks than if the program did not exist.” Do you agree with this statement?
    1. Not at all
    2. Somewhat
    3. Completely
19. The COVID-19 pandemic will increase the popularity and/or use of drug importation in this State.
    1. Disagree
    2. Neutral
    3. Agree
20. What type of pharmacy degree do you possess? Please check all that apply.
    1. B.S.
    2. M.S.
    3. Pharm.D.
    4. Ph.D.
    5. Still working on my degree (student)
    6. Other (please specify)
21. What post-graduate training have you completed or are currently working toward? Please select all that apply.
    1. Residency – PGY1
    2. Residency – PGY2
    3. Fellowship
    4. M.S.
    5. Ph.D.
    6. MBA
    7. Not applicable
    8. Other (please specify)
22. In what year did you receive your entry-level pharmacy degree?
    1. 1987 or before
    2. 1988 to 1997
    3. 1998 to 2007
    4. 2008 to 2012
    5. 2013 to 2018
23. Are you licensed in Florida?
    1. Yes
    2. No
24. Are you licensed in any other states? Please list.
25. Which of the following best describes your primary practice setting? (Please select only one).
    1. Chain pharmacy (4+ units)
    2. Supermarket pharmacy
    3. Mass-merchant pharmacy
    4. Independent pharmacy (1-3 units)
    5. Hospital/institutional (inpatient) pharmacy
    6. Clinic (outpatient) pharmacy
    7. Consultant pharmacy
    8. Mail-service pharmacy
    9. Managed care pharmacy
    10. Nuclear pharmacy
    11. Long-term care pharmacy
    12. Pharmaceutical industry
    13. Physician office-based practice
    14. Specialty pharmacy
    15. Academia (college or school of pharmacy)
    16. Ambulatory care clinic
    17. Association/regulatory
    18. Federal/military/Department of Defense pharmacy
    19. Residency/fellowship/postgraduate training
    20. Currently not working
    21. Other (please specify)
26. What is your gender?
    1. Male
    2. Female
    3. Prefer not to answer
27. What other comments or concerns do you have about this topic? [open-ended]
